# Modeling to explore and challenge inherent assumptions when cultural norms have changed: a case study on left-handedness and life expectancy

**DOI:** 10.1186/s13690-023-01156-6

**Published:** 2023-07-26

**Authors:** Juan Lavista Ferres, Md Nasir, Avleen Bijral, S V Subramanian, William B Weeks

**Affiliations:** 1 AI for Good Lab, Microsoft Corporation, Redmond, WA USA; 2 Microsoft Corporation, Redmond, WA USA; 3 Department of Social and Behavioral Sciences, Harvard T.H. Chan School of Public Health, Boston, MA USA

**Keywords:** Epidemiology, Modeling, Handedness, Life expectancy

## Abstract

**Background:**

In 1991, Halpern and Coren claimed that left-handed people die nine years younger than right-handed people. Most subsequent studies did not find support for the difference in age of death or its magnitude, primarily because of the realization that there have been historical changes in reported rates of left-handedness.

**Methods:**

We created a model that allowed us to determine whether the historical change in left-handedness explains the original finding of a nine-year difference in life expectancy. We calculated all deaths in the United States by birth year, gender, and handedness for 1989 (the Halpern and Coren study was based on data from that year) and contrasted those findings with the modeled age of death by reported and counterfactual estimated handedness for each birth year, 1900–1989.

**Results:**

In 1989, 2,019,512 individuals died, of which 6.4% were reportedly left-handed based on concurrent annual handedness reporting. However, it is widely believed that cultural pressures may have caused an underestimation of the true rate of left-handedness. Using a simulation that assumed no age of death difference between left-handed and right-handed individuals in this cohort and adjusting for the reported rates of left-handedness, we found that left-handed individuals were expected to die 9.3 years earlier than their right-handed counterparts due to changes in the rate of left-handedness over time. This difference of 9.3 years was not found to be statistically significant compared to the 8.97 years reported by Halpern and Coren. When we assumed no change in the rate of left-handedness over time, the survival advantage for right-handed individuals was reduced to 0.02 years, solely driven by not controlling for gender. When we considered the estimated age of death for each birth cohort, we found a mean difference of 0.43 years between left-handed and right-handed individuals, also driven by handedness difference by gender.

**Conclusion:**

We found that the changing rate of left-handedness reporting over the years entirely explains the originally reported observation of nine-year difference in life expectancy. In epidemiology, new information on past reporting biases could warrant re-exploration of initial findings. The simulation modeling approach that we use here might facilitate such analyses.

**Supplementary Information:**

The online version contains supplementary material available at 10.1186/s13690-023-01156-6.

## Background

In 1991, Halpern and Coren published brief [1] and more detailed [2] reports that claimed that left-handed people die nine years younger than right-handed people and suggested that left-handed people are at a higher risk of death at any given age. The authors arrived at their conclusions based on analysis of surveys sent to family members of people who died in 1989 in two southern California counties that asked about the decedents’ handedness. They attributed the nine years difference to both pathological factors and environmental interactions: the increased risk of death was likely due to correlates of left-handedness, not the left-handedness itself, as well as a potential increase in accidents due to interactions with the technological environment [1, 2].

The study was frequently cited, sometimes suggesting problems with the analysis and disagreeing with the findings [3]. Various letters to the editor were written in reply to the study, suggesting methodological problems [3–5]. One critique of a similar finding pointed out that findings of shorter lifespans in left-handed people could be explained by the fact that – because of social pressures - the percentage of left-handed people had been growing in the population over time [6].

Various subsequent studies of athletes [7–9] did not find significant differences in lifespan based on handedness, and a study of 118 same-sex twin pairs with opposite-handedness found that slightly more right-handed individuals died before their non-right-handed twin [10].

The likely confounder – that reporting of handedness changed over time, in part due to increasing cultural acceptance of left-handedness – was supported by a self-report survey of 1,177,507 National Geographic readers aged 10 to 86 that found non-right-handedness was most prevalent at younger ages (14% in men, 12% in women) and least prevalent among the elderly (6% for both men and women) and concluded that the reduction in non-right-handedness was consistent with a historical reduction in sanctions on left-handed writing early in the 20th century [11]. A later examination of survey data in the United Kingdom showed that the rate of complete left-handedness was ~ 3% for those born around 1900 and increased substantially by the mid-20th century [12].

This kind of confounding – wherein a measure as reported in real time might not reflect reality because of social or cultural normative pressures – might not be uncommon in longitudinal epidemiological studies. By using this cultural change in left-handedness reporting as an example, we developed a method that allows for comparison of mortality based on originally reported and actual (or estimated actual) data on a key variable, one that might be used more broadly in longitudinal epidemiological studies. Specifically, our three-step method uses concurrently reported estimates of the demographic factor in question (handedness as reported at the time, influenced by contemporary culture) to replicate the study in question (the Halpern-Coren study), uses counterfactual demographic data (handedness as estimated, without contemporary cultural bias) to simulate study finding estimates had the cultural bias not existed, and compares findings from the two estimates.

## Results

### Number of births, right-handed and left-handed people, and deaths in 1989

**Fig. 1:**
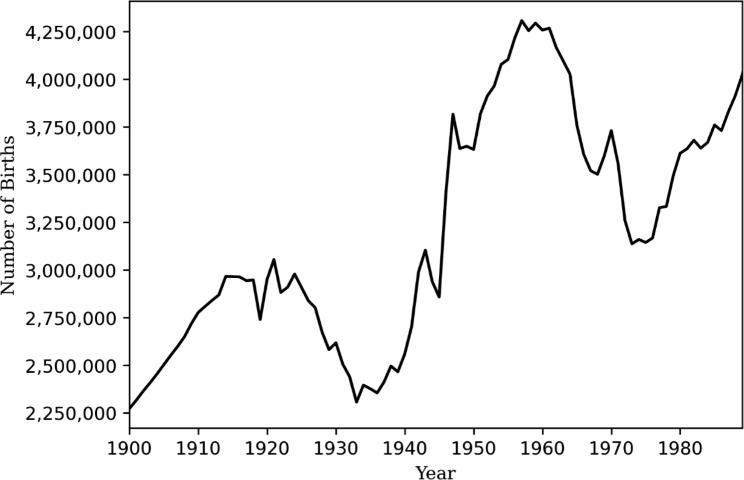
Live Births in the US by birth year, 1900–1988. Data extracted from birth data of the National Center of Health Statistics [13, 14].

The number of live births per year varied from 2,272,205 in 1900 to 4,308,000 in 1957 (Fig. 1**)**, with the cumulative number of live births from 1900 to 1988 being 284,453,782 and consisting of 143,933,614 men (50.6%) and 140,520,168 women (49.4%) (Supplemental Tables 1, left).

**Fig. 2:**
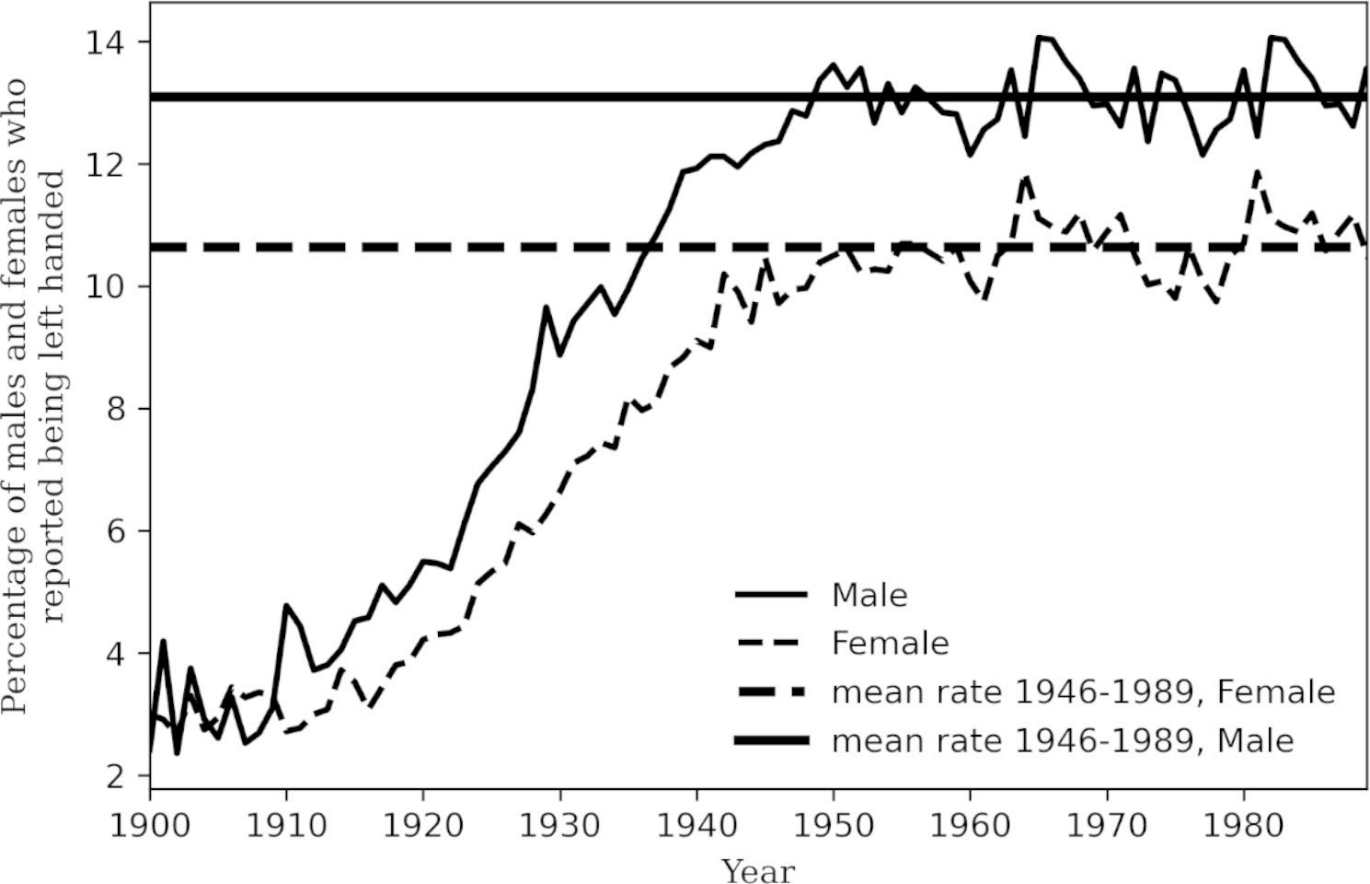
Rate of left-handed people by birth year and gender, United States, 1900–1988. Data extracted from the handedness study [12]. Horizontal lines show mean rates for each gender during the period 1946–1989

Between 1900 and 1988, the rate of concurrently reported left-handedness varied from 2.5% to 1902 to 12.6% in 1965 (Fig. 2**)**. The mean rate of left-handedness was 8.9% (9.9% (range 2.4–14.1%) for men and 7.9% (2.7–11.9%) for women) (Supplemental Tables 1, right).

We estimated that 2,019,517 people (1,043,504 men and 976,013 women) who were born between 1900 and 1988 died in 1989 (Supplemental Table 2). Assuming no handedness-related difference in mortality rates, we estimated that 1,891,554 (93.7%) were right-handed and 127,963 (6.3%) were left-handed, 77,452 of whom were men and 51,751 of whom were women (Supplemental Table 2).

### Age at death of left-handed and right-handed people in 1989

**Fig. 3:**
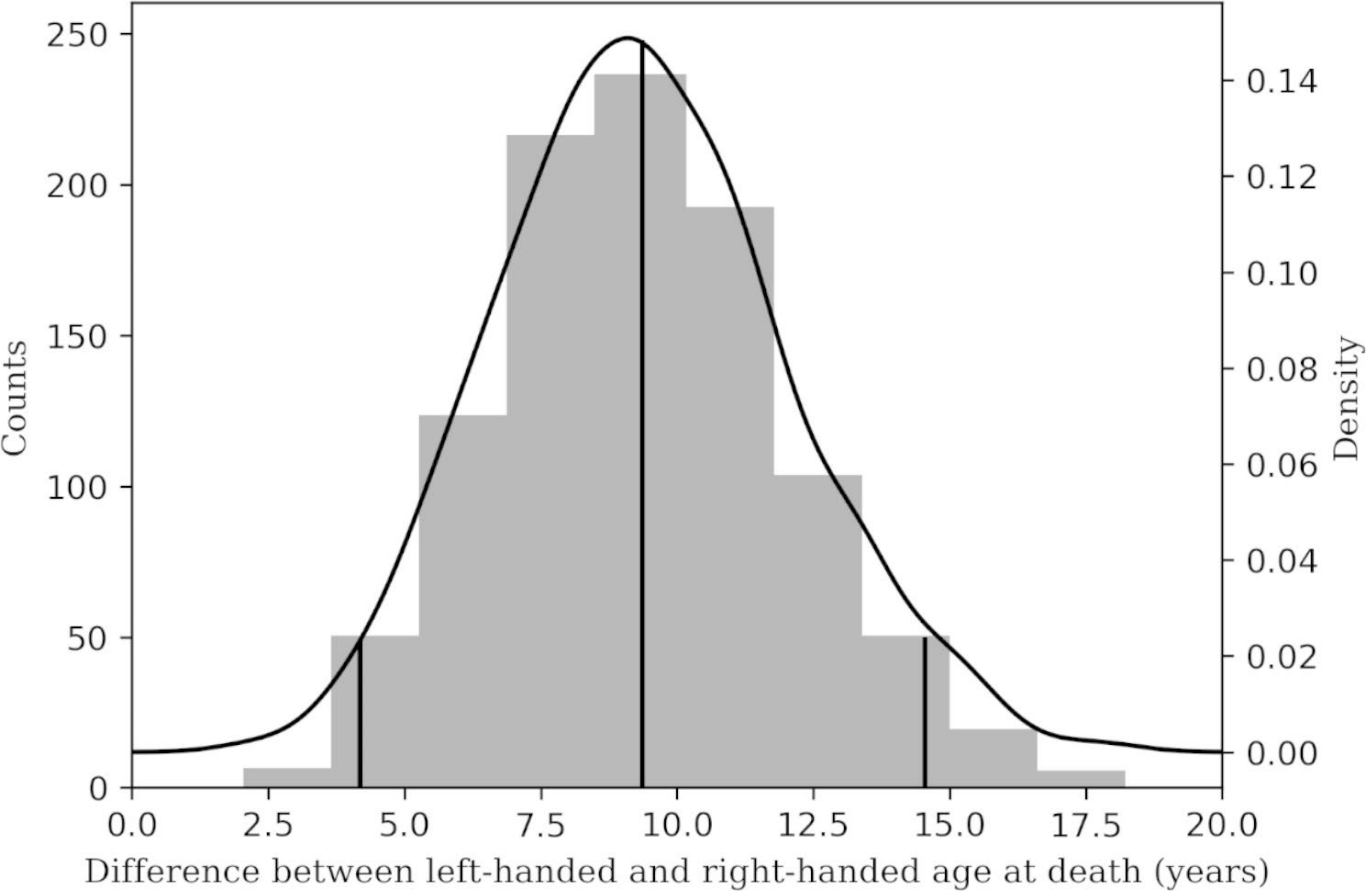
Distribution of difference in age at death of the 1989 United States death cohort. To estimate the mean difference and the confidence interval we ran 1,000 bootstraps of subsets of 989 individuals in the dataset to simulate the data from the Halpern and Coren study [1]. The left y-axis indicates the histogram counts, the right y-axis indicates the probability distribution function values (density). The black curve indicates the distribution plot fitted with the KDE method. The long vertical black line indicates the mean difference (9.3 years); the short vertical black lines indicate the confidence interval (4.26–14.40 years)

Our bootstrap analysis of 1,000 random samples of 987 deaths in 1989 found that right-handed people lived 9.3 years longer than left-handed people (95% CI: 4.3–14.4 years), (Fig. 3), results not statistically different from the 8.97 years difference reported by Halpern and Coren [1, 2] (p-value of 0.481 obtained from parametric bootstrapping).

### Age at death of left-handed and right-handed people in 1989 assuming constant handedness rates

Reported left-handedness rates were stable between 1946 and 1989. The mean left-handed rates for male and female subjects computed over this period were 13.1% and 10.6% respectively (shown with horizontal lines in Fig. 2). Assuming that these were indeed the constant rate of left-handedness for all the birth cohorts between1900-1989, we repeated our bootstrap analysis for 987 samples of population who died in 1989. Contrary to the previous analysis where we used actual reported handedness rates, we found essentially no difference (0.02 years) between the age of death of left-handed (61.82 years) and right-handed people (61.84 years).

## Discussion

We used several data sources to model the number of left- and right-handed men and women who died in the United States in 1989 and a bootstrapped sampling approach to replicate findings from Halpern and Coren, and we found similar results to theirs when not correcting for the historical change in handedness ratios. However, when appropriately controlling for handedness rates per birth cohort using two different methods, we found very little difference in longevity between left- and right-handed individuals.

In essence, our analysis reveals an example of classical data fallacy, wherein only numerators of rates are compared rather than the rates themselves, which is surprisingly common in the medical literature [15]. For instance, in articles claiming that anesthesiologists [16] and female doctors die younger than other doctors, [17] reported differences resulted from the fact that a greater proportion of anesthesiologists and women, respectively, had recently entered medical school, therefore biasing the samples [15]. Halpern and Coren dismissed from the outset the possibility that a change in left-handedness rate could have affected the results, thereby allowing the classical data fallacy to influence their findings.

Death cohort studies are challenging because they assume that the underlying ratio of.

populations is stable through the years, and this can be inaccurate [18, 19]. On the other hand, birth cohort studies solve part of this problem, though they can still be affected by confounding effects [20, 21]. When cultural norms change, similar scenarios may arise in public health cohort studies that explore, for instance, relationships between health outcomes and characteristics like gender identity, sexual orientation and behavior, reported social distancing behavior during COVID-19 pandemic, and illicit substance use where temporal variations in underlying confounding factors could be overlooked [29, 30].

This study has several limitations. The first is that estimated actual rates of handedness used were based on survey data of nearly 1.2 million readers of the National Geographic collected in 1986 [11] who were not representative of the US population in racial distribution; because there is some ethnic variation in handedness rates, [22] and we could not include data on race and ethnicity in our analysis, our findings are limited. The second limitation is that our methodology relies on a simulation of the data from Halpern and Coren’s study and hence does not represent the exact same sample. Finally, while using birth cohorts, as we did, solves some death cohort study problems, birth cohort studies can still be affected by confounders [29, 30]. Since, by design, we anticipated no difference on mortality depending on handedness, the reason for the minor difference is likely because we could not control for gender.

## Conclusions

In conclusion, we have shown that the changing rate of left-handedness over the years entirely explains the nine-year difference in life expectancy that was originally reported. Our methods might be used in other epidemiological studies wherein reported rates of a particular variable change over time, perhaps influenced by changing cultural norms.

## Methods

**Fig. 4:**
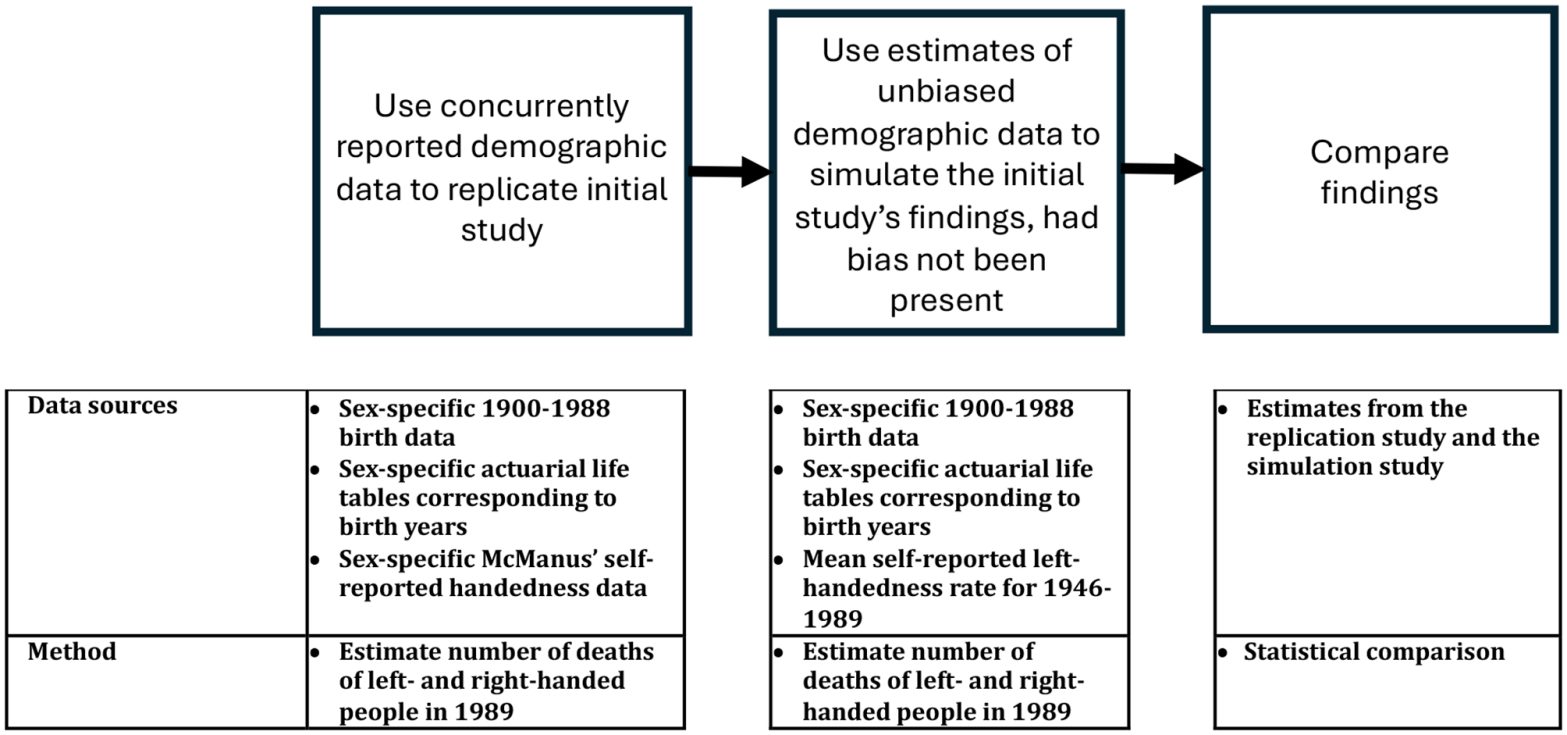
Methodology used. The overall, three-step methodology we used is outlined in the boxes at the top. Data sources and the specific methods used for the handedness example for each step are provided in the table at the bottom

Using the hypothesized relationship between handedness and longevity as an example, we developed a three-step method that (a) uses concurrently reported estimates of the demographic factor in question (handedness as reported at the time, influenced by contemporary culture) to replicate the study in question (the Halpern-Coren study), (b) uses counterfactual demographic data (handedness as estimated, without contemporary cultural bias) to simulate study finFig. 4t existed, and (c) compares findings (Fig. 4).

**Fig. 5:**
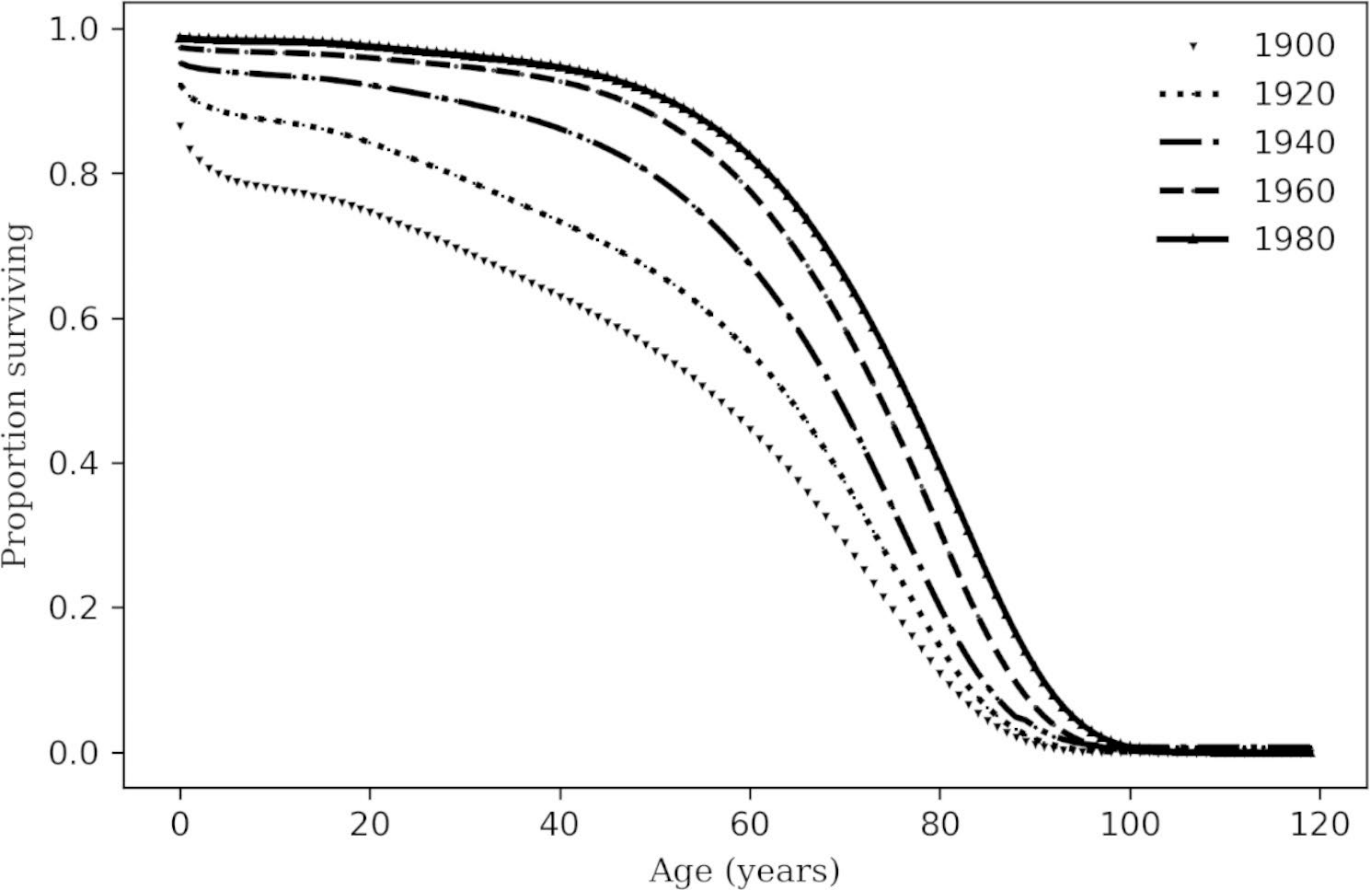
Actuarial data by 20-year birth year cohort (1900–1980), United States. Data extracted from the Actuarial Life Table [23]. While data shown combine males and females for ease of display, our analysis used gender-specific data for each cohort

To replicate findings from Halpern and Coren’s original California-based study, we generated a national estimate of the number of people that died in 1989 using three steps. First, we extracted the number of men and women born each year in the US between 1900 and 1988 from National Center of Health Statistics birth data [13, 14]. Second, we estimated the gender-specific number of births that were left- versus right-handed for each birth year from 1900 to 1988 based on McManus’ study of National Geographic readers that estimated handedness rates from 1900 to 2000 [12]. Third, under the assumption that handedness was not associated with mortality, we estimated the number of people who died in 1989 by gender and year of birth by crossing the birth data with the data from the Actuarial Life Table corresponding to birth year (Fig. 5) [23].

Given birth year (i), death year (y), handedness (h), and gender (g), we estimated the deaths as:

$$\eqalign{ Deaths[i,y,h,g] = & Births[i,g] \cr & \times Handedness[h,i,g] \cr & \times Actuarial[i,y,g] }$$

For example, according to the US birth data, [13, 14] there were 1.37 million male births in the US in 1909 (Supplemental Table 1). Based on the handedness rates study, 3.11% of those births (42,000) were left-handed males [12]. Using the Actuarial Life Table for males born in 1909, [23] we estimated that 1.5% of those males died when they were 80 years of age (Supplemental Table 2). So, assuming no handedness-related mortality differences, our model estimates that 20,774 right-handed and 667 left-handed individuals who were born in 1909 died in 1989.

### Estimated total number of right- and left-handed people who died in 1989

To arrive at an estimated total number of right- and left-handed men and women who died in 1989 and were born between 1900 and 1988, we simply summed annual estimates of deaths in 1989 by gender and handedness for those years. To simulate the sample set from Halpern and Coren that was based on 987 individuals that died in southern California in 1989, [1] we used 1,000 bootstraps of randomly selected samples of 987 from the distribution of individuals who died in 1989 and estmated 95% confidence intervals.

### Total number of right- and left-handed people that would have died in 1989, assuming constant handedness rates

In our next analysis, we considered the counterfactual scenario where the handedness rates were reported to constant (due to hypothetical absence of varying social factors). Assuming the constant rate to be the mean of the rates during the period 1946–1989, when less fluctuations were seen, we calculate estimated number deaths in 1989, both for right- and left-handed people.

### Statistical analysis

To incorporate historically changing left-handedness reporting in the United States during the period examined, we extracted the estimated rates of left-handedness from plots in McManus’s study [12] using The WebPlotDigitizer [24] tool. We used Python v3.6 and standard libraries like Pandas and NumPy [25–27] for statistical processing and data analysis. Mean estimated ages of death were compared with a student’s t-test. The kernel density estimation (KDE) method estimated the probability distribution function of the difference in age of death between right-handed and left-handed individuals [28].

## Electronic supplementary material

Below is the link to the electronic supplementary material.

Supplementary Material 1

## Data Availability

Our study utilizes publicly available open-source data. The data sample used in the study could be replicated following our methods using open source standard tools and software.
