# Supplementary material for: Modeling to explore and challenge inherent assumptions when cultural norms have changed: a case study on left-handedness and life expectancy
Source: Arch Public Health. 2023 Jul 26;81:137. doi: 10.1186/s13690-023-01156-6 (PMC10369838; doi:10.1186/s13690-023-01156-6)
Supplement: Supplementary file 1 — Supplementary Material 1 [file 13690_2023_1156_MOESM1_ESM.docx]

**Supplemental Table 1.** Number of live births and percentage of left-handedness per birth year, in total and by gender**.**

|  | **Number of live births** | | | **Left-handedness** | | |
| --- | --- | --- | --- | --- | --- | --- |
| **Birth year** | **Total** | **Males** | **Females** | **Total, %** | **Men, %** | **Women, %** |
| 1900 | 2,272,205 | 1,149,736 | 1,122,469 | 2.71 | 2.42 | 3.00 |
| 1901 | 2,316,699 | 1,172,250 | 1,144,449 | 3.55 | 4.19 | 2.91 |
| 1902 | 2,363,881 | 1,196,124 | 1,167,757 | 2.52 | 2.37 | 2.66 |
| 1903 | 2,407,778 | 1,218,336 | 1,189,442 | 3.53 | 3.75 | 3.30 |
| 1904 | 2,453,766 | 1,241,606 | 1,212,160 | 2.83 | 2.92 | 2.75 |
| 1905 | 2,503,038 | 1,266,537 | 1,236,501 | 2.78 | 2.61 | 2.94 |
| 1906 | 2,551,714 | 1,291,167 | 1,260,547 | 3.36 | 3.28 | 3.44 |
| 1907 | 2,598,298 | 1,314,739 | 1,283,559 | 2.90 | 2.53 | 3.28 |
| 1908 | 2,649,064 | 1,340,426 | 1,308,638 | 3.03 | 2.70 | 3.36 |
| 1909 | 2,718,000 | 1,375,308 | 1,342,692 | 3.21 | 3.11 | 3.30 |
| 1910 | 2,777,000 | 1,405,162 | 1,371,838 | 3.75 | 4.78 | 2.72 |
| 1911 | 2,809,000 | 1,421,354 | 1,387,646 | 3.61 | 4.44 | 2.78 |
| 1912 | 2,840,000 | 1,437,040 | 1,402,960 | 3.36 | 3.72 | 3.00 |
| 1913 | 2,869,000 | 1,451,714 | 1,417,286 | 3.44 | 3.81 | 3.08 |
| 1914 | 2,966,000 | 1,500,796 | 1,465,204 | 3.89 | 4.06 | 3.72 |
| 1915 | 2,965,000 | 1,500,290 | 1,464,710 | 4.03 | 4.53 | 3.53 |
| 1916 | 2,964,000 | 1,499,784 | 1,464,216 | 3.83 | 4.58 | 3.08 |
| 1917 | 2,944,000 | 1,489,664 | 1,454,336 | 4.28 | 5.11 | 3.44 |
| 1918 | 2,948,000 | 1,491,688 | 1,456,312 | 4.32 | 4.83 | 3.80 |
| 1919 | 2,740,000 | 1,386,440 | 1,353,560 | 4.48 | 5.11 | 3.86 |
| 1920 | 2,950,000 | 1,492,700 | 1,457,300 | 4.86 | 5.50 | 4.22 |
| 1921 | 3,055,000 | 1,545,830 | 1,509,170 | 4.89 | 5.47 | 4.30 |
| 1922 | 2,882,000 | 1,458,292 | 1,423,708 | 4.86 | 5.39 | 4.33 |
| 1923 | 2,910,000 | 1,472,460 | 1,437,540 | 5.27 | 6.11 | 4.44 |
| 1924 | 2,979,000 | 1,507,374 | 1,471,626 | 5.95 | 6.77 | 5.14 |
| 1925 | 2,909,000 | 1,471,954 | 1,437,046 | 6.19 | 7.05 | 5.33 |
| 1926 | 2,839,000 | 1,436,534 | 1,402,466 | 6.38 | 7.30 | 5.47 |
| 1927 | 2,802,000 | 1,417,812 | 1,384,188 | 6.86 | 7.60 | 6.11 |
| 1928 | 2,674,000 | 1,353,044 | 1,320,956 | 7.15 | 8.32 | 5.97 |
| 1929 | 2,582,000 | 1,306,492 | 1,275,508 | 7.97 | 9.65 | 6.28 |
| 1930 | 2,618,000 | 1,324,708 | 1,293,292 | 7.76 | 8.88 | 6.64 |
| 1931 | 2,506,000 | 1,268,036 | 1,237,964 | 8.27 | 9.43 | 7.11 |
| 1932 | 2,440,000 | 1,234,640 | 1,205,360 | 8.47 | 9.71 | 7.22 |
| 1933 | 2,307,000 | 1,167,342 | 1,139,658 | 8.71 | 9.99 | 7.44 |
| 1934 | 2,396,000 | 1,212,376 | 1,183,624 | 8.45 | 9.54 | 7.36 |
| 1935 | 2,377,000 | 1,202,762 | 1,174,238 | 9.08 | 9.96 | 8.19 |
| 1936 | 2,355,000 | 1,191,630 | 1,163,370 | 9.21 | 10.46 | 7.97 |
| 1937 | 2,413,000 | 1,220,978 | 1,192,022 | 9.45 | 10.82 | 8.08 |
| 1938 | 2,496,000 | 1,262,976 | 1,233,024 | 9.96 | 11.26 | 8.67 |
| 1939 | 2,466,000 | 1,247,796 | 1,218,204 | 10.35 | 11.87 | 8.83 |
| 1940 | 2,559,000 | 1,294,854 | 1,264,146 | 10.52 | 11.93 | 9.11 |
| 1941 | 2,703,000 | 1,367,718 | 1,335,282 | 10.56 | 12.12 | 9.00 |
| 1942 | 2,989,000 | 1,512,434 | 1,476,566 | 11.16 | 12.12 | 10.19 |
| 1943 | 3,104,000 | 1,570,624 | 1,533,376 | 10.93 | 11.95 | 9.92 |
| 1944 | 2,939,000 | 1,487,134 | 1,451,866 | 10.80 | 12.18 | 9.42 |
| 1945 | 2,858,000 | 1,446,148 | 1,411,852 | 11.39 | 12.31 | 10.47 |
| 1946 | 3,411,000 | 1,725,966 | 1,685,034 | 11.05 | 12.37 | 9.72 |
| 1947 | 3,817,000 | 1,931,402 | 1,885,598 | 11.41 | 12.87 | 9.94 |
| 1948 | 3,637,000 | 1,840,322 | 1,796,678 | 11.38 | 12.79 | 9.97 |
| 1949 | 3,649,000 | 1,846,394 | 1,802,606 | 11.88 | 13.37 | 10.39 |
| 1950 | 3,632,000 | 1,837,792 | 1,794,208 | 12.06 | 13.62 | 10.50 |
| 1951 | 3,820,000 | 1,932,920 | 1,887,080 | 11.93 | 13.26 | 10.61 |
| 1952 | 3,913,000 | 1,979,978 | 1,933,022 | 11.89 | 13.56 | 10.22 |
| 1953 | 3,965,000 | 2,006,290 | 1,958,710 | 11.48 | 12.67 | 10.28 |
| 1954 | 4,078,000 | 2,063,468 | 2,014,532 | 11.78 | 13.31 | 10.25 |
| 1955 | 4,104,000 | 2,076,624 | 2,027,376 | 11.77 | 12.84 | 10.69 |
| 1956 | 4,218,000 | 2,134,308 | 2,083,692 | 11.98 | 13.26 | 10.69 |
| 1957 | 4,308,000 | 2,179,848 | 2,128,152 | 11.81 | 13.06 | 10.55 |
| 1958 | 4,255,000 | 2,153,030 | 2,101,970 | 11.63 | 12.84 | 10.42 |
| 1959 | 4,295,000 | 2,173,270 | 2,121,730 | 11.73 | 12.81 | 10.64 |
| 1960 | 4,257,850 | 2,154,472 | 2,103,378 | 11.12 | 12.15 | 10.08 |
| 1961 | 4,268,326 | 2,159,773 | 2,108,553 | 11.16 | 12.56 | 9.75 |
| 1962 | 4,167,362 | 2,108,685 | 2,058,677 | 11.61 | 12.73 | 10.50 |
| 1963 | 4,098,020 | 2,073,598 | 2,024,422 | 12.11 | 13.53 | 10.69 |
| 1964 | 4,027,490 | 2,037,910 | 1,989,580 | 12.16 | 12.45 | 11.86 |
| 1965 | 3,760,358 | 1,902,741 | 1,857,617 | 12.59 | 14.06 | 11.11 |
| 1966 | 3,606,274 | 1,824,775 | 1,781,499 | 12.50 | 14.03 | 10.97 |
| 1967 | 3,520,959 | 1,781,605 | 1,739,354 | 12.28 | 13.67 | 10.89 |
| 1968 | 3,501,564 | 1,771,791 | 1,729,773 | 12.29 | 13.39 | 11.19 |
| 1969 | 3,600,206 | 1,821,704 | 1,778,502 | 11.77 | 12.95 | 10.58 |
| 1970 | 3,731,386 | 1,888,081 | 1,843,305 | 11.93 | 12.98 | 10.89 |
| 1971 | 3,555,970 | 1,799,321 | 1,756,649 | 11.89 | 12.62 | 11.17 |
| 1972 | 3,258,411 | 1,648,756 | 1,609,655 | 12.04 | 13.56 | 10.53 |
| 1973 | 3,136,965 | 1,587,304 | 1,549,661 | 11.20 | 12.37 | 10.03 |
| 1974 | 3,159,958 | 1,598,939 | 1,561,019 | 11.78 | 13.48 | 10.08 |
| 1975 | 3,144,198 | 1,590,964 | 1,553,234 | 11.59 | 13.37 | 9.80 |
| 1976 | 3,167,788 | 1,602,901 | 1,564,887 | 11.73 | 12.81 | 10.64 |
| 1977 | 3,326,632 | 1,683,276 | 1,643,356 | 11.12 | 12.15 | 10.08 |
| 1978 | 3,333,279 | 1,686,639 | 1,646,640 | 11.16 | 12.56 | 9.75 |
| 1979 | 3,494,398 | 1,768,165 | 1,726,233 | 11.61 | 12.73 | 10.50 |
| 1980 | 3,612,258 | 1,827,803 | 1,784,455 | 12.11 | 13.53 | 10.69 |
| 1981 | 3,635,515 | 1,839,571 | 1,795,944 | 12.16 | 12.45 | 11.86 |
| 1982 | 3,680,537 | 1,862,352 | 1,818,185 | 12.59 | 14.06 | 11.11 |
| 1983 | 3,638,933 | 1,841,300 | 1,797,633 | 12.50 | 14.03 | 10.97 |
| 1984 | 3,669,141 | 1,856,585 | 1,812,556 | 12.28 | 13.67 | 10.89 |
| 1985 | 3,760,561 | 1,902,844 | 1,857,717 | 12.29 | 13.39 | 11.19 |
| 1986 | 3,731,000 | 1,887,886 | 1,843,114 | 11.77 | 12.95 | 10.58 |
| 1987 | 3,829,000 | 1,937,474 | 1,891,526 | 11.93 | 12.98 | 10.89 |
| 1988 | 3,913,000 | 1,979,978 | 1,933,022 | 11.89 | 12.62 | 11.17 |
| **Total** | **284,453,782** | **143,933,614** | **140,520,168** | NA | NA | NA |
| **Percentage** | **100.0%** | **50.6%** | **49.4%** | NA | NA | NA |
| **Mean** | NA | NA | NA | **8.92** | **9.90** | **7.94** |

NA, not applicable

## **Supplemental Table 2.** Estimated total number of people and number of left-handed people that died in 1989 by birth year, by gender.

|  | **Number of people**  **that died in 1989** | | | **Number of left-handed people**  **that died in 1989** | | |
| --- | --- | --- | --- | --- | --- | --- |
| **Birth year** | **Total** | **Men** | **Women** | **Total** | **Men** | **Women** |
| 1900 | 11,999 | 5,174 | 6,825 | 325.2 | 125.2 | 204.8 |
| 1901 | 14,746 | 6,506 | 8,240 | 523.5 | 272.6 | 239.8 |
| 1902 | 17,812 | 8,026 | 9,786 | 448.9 | 190.2 | 260.3 |
| 1903 | 21,224 | 9,710 | 11,514 | 749.2 | 364.1 | 380.0 |
| 1904 | 25,039 | 11,572 | 13,467 | 708.6 | 337.9 | 370.3 |
| 1905 | 29,268 | 13,577 | 15,691 | 813.7 | 354.4 | 461.3 |
| 1906 | 33,751 | 15,675 | 18,076 | 1134.0 | 514.1 | 621.8 |
| 1907 | 38,119 | 17,723 | 20,396 | 1105.5 | 448.4 | 669.0 |
| 1908 | 42,015 | 19,637 | 22,378 | 1273.1 | 530.2 | 751.9 |
| 1909 | 45,435 | 21,441 | 23,994 | 1458.5 | 666.8 | 791.8 |
| 1910 | 54,040 | 25,012 | 29,028 | 2026.5 | 1195.6 | 789.6 |
| 1911 | 56,154 | 26,181 | 29,973 | 2027.2 | 1162.4 | 833.2 |
| 1912 | 57,829 | 27,146 | 30,683 | 1943.1 | 1009.8 | 920.5 |
| 1913 | 59,398 | 28,076 | 31,322 | 2043.3 | 1069.7 | 964.7 |
| 1914 | 62,374 | 29,671 | 32,703 | 2426.3 | 1204.6 | 1216.6 |
| 1915 | 63,054 | 30,186 | 32,868 | 2541.1 | 1367.4 | 1160.2 |
| 1916 | 63,260 | 30,476 | 32,784 | 2422.9 | 1395.8 | 1009.7 |
| 1917 | 62,528 | 30,300 | 32,228 | 2676.2 | 1548.3 | 1108.6 |
| 1918 | 61,660 | 30,043 | 31,617 | 2663.7 | 1451.1 | 1201.4 |
| 1919 | 55,874 | 27,354 | 28,520 | 2503.2 | 1397.8 | 1100.9 |
| 1920 | 61,839 | 31,600 | 30,239 | 3005.4 | 1738.0 | 1276.1 |
| 1921 | 61,735 | 31,612 | 30,123 | 3018.8 | 1729.2 | 1295.3 |
| 1922 | 55,834 | 28,641 | 27,193 | 2713.5 | 1543.7 | 1177.5 |
| 1923 | 53,729 | 27,609 | 26,120 | 2831.5 | 1686.9 | 1159.7 |
| 1924 | 52,143 | 26,831 | 25,312 | 3102.5 | 1816.5 | 1301.0 |
| 1925 | 48,081 | 24,758 | 23,323 | 2976.2 | 1745.4 | 1243.1 |
| 1926 | 44,241 | 22,783 | 21,458 | 2822.6 | 1663.2 | 1173.8 |
| 1927 | 41,298 | 21,296 | 20,002 | 2833.0 | 1618.5 | 1222.1 |
| 1928 | 37,513 | 19,389 | 18,124 | 2682.2 | 1613.2 | 1082.0 |
| 1929 | 34,686 | 17,990 | 16,696 | 2764.5 | 1736.0 | 1048.5 |
| 1930 | 37,940 | 20,506 | 17,434 | 2944.1 | 1820.9 | 1157.6 |
| 1931 | 34,412 | 18,665 | 15,747 | 2845.9 | 1760.1 | 1119.6 |
| 1932 | 31,773 | 17,285 | 14,488 | 2691.2 | 1678.4 | 1046.0 |
| 1933 | 28,554 | 15,596 | 12,958 | 2487.1 | 1558.0 | 964.1 |
| 1934 | 28,230 | 15,482 | 12,748 | 2385.4 | 1477.0 | 938.3 |
| 1935 | 26,734 | 14,722 | 12,012 | 2427.4 | 1466.3 | 983.8 |
| 1936 | 25,274 | 13,966 | 11,308 | 2327.7 | 1460.8 | 901.2 |
| 1937 | 24,677 | 13,675 | 11,002 | 2332.0 | 1479.6 | 889.0 |
| 1938 | 24,240 | 13,451 | 10,789 | 2414.3 | 1514.6 | 935.4 |
| 1939 | 22,640 | 12,565 | 10,075 | 2343.2 | 1491.5 | 889.6 |
| 1940 | 20,715 | 12,094 | 8,621 | 2179.2 | 1442.8 | 785.4 |
| 1941 | 20,418 | 11,899 | 8,519 | 2156.1 | 1442.2 | 766.7 |
| 1942 | 21,081 | 12,251 | 8,830 | 2352.6 | 1484.8 | 899.8 |
| 1943 | 20,507 | 11,874 | 8,633 | 2241.4 | 1418.9 | 856.4 |
| 1944 | 18,253 | 10,529 | 7,724 | 1971.3 | 1282.4 | 727.6 |
| 1945 | 16,718 | 9,588 | 7,130 | 1904.2 | 1180.3 | 746.5 |
| 1946 | 18,806 | 10,718 | 8,088 | 2078.1 | 1325.8 | 786.2 |
| 1947 | 19,820 | 11,241 | 8,579 | 2261.5 | 1446.7 | 852.8 |
| 1948 | 17,737 | 10,011 | 7,726 | 2018.5 | 1280.4 | 770.3 |
| 1949 | 16,662 | 9,361 | 7,301 | 1979.4 | 1251.6 | 758.6 |
| 1950 | 11,173 | 6,598 | 4,575 | 1347.5 | 898.6 | 480.4 |
| 1951 | 10,794 | 6,340 | 4,454 | 1287.7 | 840.7 | 472.6 |
| 1952 | 10,194 | 5,980 | 4,214 | 1212.1 | 810.9 | 430.7 |
| 1953 | 9,555 | 5,598 | 3,957 | 1096.9 | 709.3 | 406.8 |
| 1954 | 9,131 | 5,344 | 3,787 | 1075.6 | 711.3 | 388.2 |
| 1955 | 8,594 | 5,046 | 3,548 | 1011.5 | 647.9 | 379.3 |
| 1956 | 8,284 | 4,888 | 3,396 | 992.4 | 648.1 | 363.0 |
| 1957 | 7,986 | 4,730 | 3,256 | 943.1 | 617.7 | 343.5 |
| 1958 | 7,484 | 4,457 | 3,027 | 870.4 | 572.3 | 315.4 |
| 1959 | 7,189 | 4,303 | 2,886 | 843.3 | 551.2 | 307.1 |
| 1960 | 5,617 | 3,598 | 2,019 | 624.6 | 437.2 | 203.5 |
| 1961 | 5,418 | 3,520 | 1,898 | 604.6 | 442.1 | 185.1 |
| 1962 | 5,186 | 3,416 | 1,770 | 602.1 | 434.9 | 185.9 |
| 1963 | 5,041 | 3,401 | 1,640 | 610.5 | 460.2 | 175.3 |
| 1964 | 4,935 | 3,403 | 1,532 | 600.1 | 423.7 | 181.7 |
| 1965 | 4,648 | 3,273 | 1,375 | 585.2 | 460.2 | 152.8 |
| 1966 | 4,440 | 3,193 | 1,247 | 555.0 | 448.0 | 136.8 |
| 1967 | 4,283 | 3,118 | 1,165 | 526.0 | 426.2 | 126.9 |
| 1968 | 4,101 | 2,994 | 1,107 | 504.0 | 400.9 | 123.9 |
| 1969 | 4,000 | 2,915 | 1,085 | 470.8 | 377.5 | 114.8 |
| 1970 | 4,803 | 3,550 | 1,253 | 573.0 | 460.8 | 136.5 |
| 1971 | 4,290 | 3,131 | 1,159 | 510.1 | 395.1 | 129.5 |
| 1972 | 3,587 | 2,589 | 998 | 431.9 | 351.1 | 105.1 |
| 1973 | 2,995 | 2,127 | 868 | 335.4 | 263.1 | 87.1 |
| 1974 | 2,477 | 1,743 | 734 | 291.8 | 235.0 | 74.0 |
| 1975 | 1,911 | 1,305 | 606 | 221.5 | 174.5 | 59.4 |
| 1976 | 1,415 | 930 | 485 | 166.0 | 119.1 | 51.6 |
| 1977 | 1,117 | 690 | 427 | 124.2 | 83.8 | 43.0 |
| 1978 | 968 | 573 | 395 | 108.0 | 72.0 | 38.5 |
| 1979 | 1,033 | 601 | 432 | 119.9 | 76.5 | 45.4 |
| 1980 | 869 | 494 | 375 | 105.2 | 66.8 | 40.1 |
| 1981 | 1,020 | 589 | 431 | 124.0 | 73.3 | 51.1 |
| 1982 | 1,143 | 670 | 473 | 143.9 | 94.2 | 52.6 |
| 1983 | 1,221 | 718 | 503 | 152.6 | 100.7 | 55.2 |
| 1984 | 1,287 | 761 | 526 | 158.0 | 104.0 | 57.3 |
| 1985 | 1,451 | 875 | 576 | 178.3 | 117.2 | 64.5 |
| 1986 | 1,870 | 1,114 | 756 | 220.1 | 144.3 | 80.0 |
| 1987 | 2,416 | 1,376 | 1,040 | 288.2 | 178.6 | 113.3 |
| 1988 | 3,722 | 2,079 | 1,643 | 442.5 | 262.4 | 183.5 |
| **Total** | **2,019,517** | **1,043,504** | **976,013** | **127,962.6** | **77,451.7** | **51,751.1** |

## **Supplemental Table 3.** Modeled difference in age at death in years based on handedness per birth year, in total and by gender.

|  | **Difference in age at death (in years) based on handedness** | | |
| --- | --- | --- | --- |
| **Birth year** | **Total** | **Men** | **Women** |
| 1900 | -0.21 | -0.31 | 0.13 |
| 1901 | -0.24 | -0.41 | -0.61 |
| 1902 | 0.26 | 0.71 | -0.03 |
| 1903 | 0.35 | 0.26 | 0.26 |
| 1904 | 0.33 | 0.41 | 0.13 |
| 1905 | -0.37 | -0.29 | -0.27 |
| 1906 | -0.10 | 0.03 | -0.19 |
| 1907 | -0.02 | 0.42 | -0.06 |
| 1908 | -0.29 | -0.02 | -0.28 |
| 1909 | -0.09 | 0.00 | -0.11 |
| 1910 | 0.47 | -0.21 | 0.31 |
| 1911 | 0.66 | 0.18 | 0.33 |
| 1912 | 0.28 | -0.30 | 0.56 |
| 1913 | 0.18 | -0.02 | -0.02 |
| 1914 | 0.02 | -0.24 | 0.14 |
| 1915 | -0.06 | -0.24 | -0.33 |
| 1916 | 0.32 | -0.04 | 0.00 |
| 1917 | 0.37 | 0.03 | -0.01 |
| 1918 | 0.02 | 0.04 | -0.51 |
| 1919 | 0.28 | 0.27 | -0.30 |
| 1920 | 0.19 | 0.05 | 0.09 |
| 1921 | -0.12 | -0.37 | -0.05 |
| 1922 | 0.00 | -0.18 | -0.04 |
| 1923 | 0.10 | 0.01 | -0.13 |
| 1924 | 0.20 | 0.18 | -0.05 |
| 1925 | 0.05 | -0.31 | 0.22 |
| 1926 | -0.09 | -0.24 | -0.21 |
| 1927 | 0.14 | -0.05 | 0.13 |
| 1928 | -0.04 | -0.26 | -0.11 |
| 1929 | 0.12 | -0.06 | -0.12 |
| 1930 | 0.43 | 0.14 | 0.19 |
| 1931 | 0.21 | 0.22 | -0.36 |
| 1932 | 0.25 | -0.01 | -0.01 |
| 1933 | 0.07 | -0.21 | -0.13 |
| 1934 | 0.26 | -0.11 | 0.21 |
| 1935 | 0.26 | -0.02 | 0.19 |
| 1936 | 0.17 | -0.20 | 0.09 |
| 1937 | 0.28 | 0.03 | -0.03 |
| 1938 | 0.37 | 0.14 | 0.10 |
| 1939 | 0.36 | -0.06 | 0.27 |
| 1940 | 0.47 | 0.29 | 0.07 |
| 1941 | 0.35 | 0.10 | -0.10 |
| 1942 | 0.16 | -0.09 | 0.00 |
| 1943 | 0.19 | -0.23 | 0.19 |
| 1944 | 0.39 | 0.09 | 0.13 |
| 1945 | 0.23 | 0.12 | -0.04 |
| 1946 | 0.22 | 0.06 | -0.16 |
| 1947 | 0.27 | -0.20 | 0.20 |
| 1948 | 0.34 | -0.05 | 0.22 |
| 1949 | 0.34 | 0.00 | 0.15 |
| 1950 | 0.43 | -0.03 | 0.11 |
| 1951 | 0.39 | 0.05 | 0.03 |
| 1952 | 0.40 | 0.03 | -0.10 |
| 1953 | 0.27 | -0.01 | -0.12 |
| 1954 | 0.36 | 0.04 | -0.15 |
| 1955 | 0.40 | 0.15 | 0.11 |
| 1956 | 0.26 | -0.01 | -0.15 |
| 1957 | 0.42 | 0.16 | -0.01 |
| 1958 | 0.23 | -0.13 | -0.07 |
| 1959 | 0.25 | -0.03 | -0.09 |
| 1960 | 0.38 | 0.13 | -0.04 |
| 1961 | 0.50 | 0.04 | 0.05 |
| 1962 | 0.38 | -0.03 | 0.09 |
| 1963 | 0.42 | 0.03 | -0.09 |
| 1964 | 0.14 | 0.00 | 0.08 |
| 1965 | 0.46 | 0.00 | 0.00 |
| 1966 | 0.39 | -0.08 | -0.06 |
| 1967 | 0.33 | -0.14 | -0.06 |
| 1968 | 0.19 | -0.27 | 0.00 |
| 1969 | 0.39 | -0.09 | 0.10 |
| 1970 | 0.45 | 0.15 | -0.02 |
| 1971 | 0.27 | 0.12 | -0.10 |
| 1972 | 0.59 | 0.01 | 0.11 |
| 1973 | 0.61 | 0.10 | 0.18 |
| 1974 | 0.69 | 0.11 | 0.00 |
| 1975 | 0.76 | -0.02 | 0.28 |
| 1976 | 0.47 | -0.03 | 0.20 |
| 1977 | 0.29 | -0.11 | -0.09 |
| 1978 | 0.60 | -0.09 | 0.23 |
| 1979 | 0.36 | -0.11 | -0.02 |
| 1980 | 0.54 | 0.01 | 0.10 |
| 1981 | 0.14 | -0.02 | 0.14 |
| 1982 | 0.50 | -0.02 | -0.01 |
| 1983 | 0.45 | -0.02 | -0.13 |
| 1984 | 0.47 | 0.10 | -0.10 |
| 1985 | 0.45 | 0.09 | -0.04 |
| 1986 | 0.53 | 0.06 | 0.15 |
| 1987 | 0.41 | -0.06 | 0.16 |
| 1988 | 0.16 | -0.11 | -0.11 |
